# Identification of GLS as a cuproptosis-related diagnosis gene in acute myocardial infarction

**DOI:** 10.3389/fcvm.2022.1016081

**Published:** 2022-11-11

**Authors:** Zheng Liu, Lei Wang, Qichang Xing, Xiang Liu, Yixiang Hu, Wencan Li, Qingzi Yan, Renzhu Liu, Nan Huang

**Affiliations:** ^1^Clinical Pharmacy, Xiangtan Center Hospital, Xiangtan, China; ^2^Zhou Honghao Research Institute Xiangtan, Xiangtan, China; ^3^Department of Cardiovascular Medicine, Xiangtan Center Hospital, Xiangtan, China

**Keywords:** cuproptosis-related genes, acute myocardial infarction, immune landscape analysis, GLS, GSEA

## Abstract

Acute myocardial infarction (AMI) has the characteristics of sudden onset, rapid progression, poor prognosis, and so on. Therefore, it is urgent to identify diagnostic and prognostic biomarkers for it. Cuproptosis is a new form of mitochondrial respiratory-dependent cell death. However, studies are limited on the clinical significance of cuproptosis-related genes (CRGs) in AMI. In this study, we systematically assessed the genetic alterations of CRGs in AMI by bioinformatics approach. The results showed that six CRGs (LIAS, LIPT1, DLAT, PDHB, MTF1, and GLS) were markedly differentially expressed between stable coronary heart disease (stable_CAD) and AMI. Correlation analysis indicated that CRGs were closely correlated with N6-methyladenosine (m6A)-related genes through R language “corrplot” package, especially GLS was positively correlated with FMR1 and MTF1 was negatively correlated with HNRNPA2B1. Immune landscape analysis results revealed that CRGs were closely related to various immune cells, especially GLS was positively correlated with T cells CD4 memory resting and negatively correlated with monocytes. Kaplan–Meier analysis demonstrated that the group with high DLAT expression had a better prognosis. The area under curve (AUC) certified that GLS had good diagnostic value, in the training set (AUC = 0.87) and verification set (ACU = 0.99). Gene set enrichment analysis (GSEA) suggested that GLS was associated with immune- and hypoxia-related pathways. In addition, Gene Ontology (GO) analysis, Kyoto Encyclopedia of Genes and Genomes (KEGG) analysis, competing endogenous RNA (ceRNA) analysis, transcription factor (TF), and compound prediction were performed to reveal the regulatory mechanism of CRGs in AMI. Overall, our study can provide additional information for understanding the role of CRGs in AMI, which may provide new insights into the identification of therapeutic targets for AMI.

## Introduction

Acute myocardial infarction (AMI) is a cardiovascular disease which not only endangers human health but also endangers human life (1). In recent decades, with the increase in evidence-based therapy and lifestyle interventions, the incidence of coronary heart disease has declined significantly (2). Nevertheless, AMI still seriously threatens to global health, affecting more than seven million people worldwide each year. Notably, while the incidence of AMI declined in older patients, there was no similar reduction in younger patients (3). Patients with AMI may typically have persistent chest pain, or persistent shortness of breath, sweating, nausea, unexplained weakness, or a combination of these symptoms (4). The current therapy strategy for AMI is to restore coronary blood flow (reperfusion) through implantation of endovascular stents (4), although clinical reperfusion therapy can protect the heart from further damage and enhance the risk of short-term and long-term heart failure caused by AMI, which presents treatment challenges (5). The identification of AMI biomarkers will play an important role in early diagnosis.

Acute myocardial infarction is one of the progressive outcomes of atherosclerosis (6). The destruction of the intima of the vessel results in lipid accumulation, accompanied by a large number of immune cell infiltration (7). Inflammatory factors produced by immune cells stimulate the proliferation and migration of smooth muscle cells into the intima (8–10). Progressively, fibrous caps, principally composed of calcified smooth muscle, form in distinct microenvironments (11, 12). In addition, necrotizing cells appear in the plaque area as immune cells continue to infiltrate (13). A large number of necrotic cells lead to a thinning of the fibrous cap, which in turn lead to plaque instability. Plaque ruptures can cause coronary artery thrombosis, which triggers AMI (14). At the same time, cardiomyocyte necrosis occurs in the area of obstruction due to hypoxia, leading to life-threatening cardiovascular events.

Copper can coordinate a variety of cellular biological processes, such as lipolysis, cell proliferation, autophagy, and neural activity (15). Interference of copper dynamic equilibrium leads to Menkes and Wilson disease (16). Noteworthy, Tsvetkov and colleagues demonstrated that a novel copper-dependent mode of cell death is independent of apoptosis, pyroptosis, and ferroptosis (17). Mitochondrial stress, characterized by excessive accumulation of lipoylated mitochondrial enzymes and depletion of Fe-S cluster proteins, has been reported to be the main mechanism leading to cuproptosis. Cupric ions can be reduced to cuprous ions by FDX1 within the mitochondrial matrix, which in turn promotes the lipoacylation of mitochondrial proteins and the overproduction of key enzymes associated with the mitochondrial tricarboxylic acid (TCA) cycle. In addition, key genes related to cupric ions transport may play an important role in regulating the occurrence of copper poisoning (17, 18). Furthermore, copper death participates in the regulation of aseptic inflammation, thereby affecting the immune microenvironment of the lesion (19). More and more evidence indicates that mitochondrial dysfunction and immune regulation are involved in the development of AMI. Therefore, we hypothesized that cuproptosis may be involved in the regulation of AMI (20, 21), but the role of CRGs in AMI is unclear. In this research, the diagnostic and prognostic values of CRGs in AMI were analyzed by bioinformatics methods, and the correlation of these genes with m6A and immune infiltration was discussed.

## Materials and methods

### Data preprocess

The data sets were from the Gene Expression Omnibus database^1^ (GEO). GSE59867 (22) was used to analyze the differences of CRGs and construct diagnostic model as a training set, including 111 patients with AMI and 46 control patients with stable_CAD and without history of AMI (Table 1). GSE62646 (23) was the validation set of the diagnostic model (Table 1). GSE21545 (24) was employed to explore the prognostic value of CRGs after AMI, including 76 patients without an ischemic event and 21 patients with an ischemic event (Table 1). The CRGs were extracted from previously published articles (17), while “LDL” was deleted because there were no related data in the above data sets. GSE184073 (25), a single-cell RNA sequence of human coronary plaque, was obtained from the GEO database to analyze the expression of GLS in single cells.

**TABLE 1:** Data sets implemented for analysis.

Data set	Gene number	Platform	Case samples	Control samples
GSE59867	23298	GPL6244	111	46
GSE62646	25293	GPL6244	14	28
GSE21545	22854	GPL570	76	21
GSE184073	23320	GPL24676	1	1

### Protein–protein interaction and correlation analysis of cuproptosis-related genes

The PPI network was constructed through the STRING database^2^ (26). The correlation of CRGs was analyzed by the “corrplot” package (R v4.1.2).

### Functional enrichment analysis of cuproptosis-related genes

Cuproptosis-related genes were executed GO analysis and KEGG pathway analysis through the “clusterProfiler 4.0” package (27), and the top 10 results were visualized by R 4.1.2 software.

### Correlation analysis between cuproptosis-related genes and N6-methyladenosine related genes

The differential expression of m6A-related genes was investigated between the stable_CAD and AMI groups by the Wilcoxon test. *P* < 0.05 was considered as a statistically significant difference. The correlation was analyzed by the “corrplot” package between CRGs and m6A-related genes.

### Correlation analysis between cuproptosis-related genes and immune characteristics

The cell-type identification by estimating relative subsets of RNA transcripts (CIBERSORT) was used to calculate the abundance of particular infiltrating immunocytes, which is an R/web version tool for deconvolution of expression matrices of human immune cell subtypes, based on linear support vector regression (28). The differential abundance of immune cell infiltration was investigated between the stable_CAD and AMI groups by the Wilcoxon test. *P* < 0.05 was considered as a statistically significant difference. The correlation between CRGs and immune cell infiltration was calculated by the Pearson correlation coefficient.

### The construction of competing endogenous RNA, transcription factor analysis, and tiny molecule drug recognition

NetworkAnalyst^3^ has been developed as a comprehensive gene-centric platform supporting gene expression profiling analysis, biological network analysis, and visual exploration (29). In our study, it was utilized for ceRNA network construction, TF analysis, and tiny molecule identification of CRGs.

### Prognostic and diagnostic values of cuproptosis-related genes

GSE21545 was employed to analyze the prognostic value of CRGs after AMI by Kaplan–Meier curves, and log-rank *P* < 0.05 indicated a significant difference between the two groups. Univariate and multivariate logistic regression combined with Lasso regression analyses were used to evaluate the diagnostic value of CRGs by R language “survival” and “glmnet” packages based on the GSE59867 data set. The diagnostic value of GLS expression in AMI patients was evaluated by the receiver operating characteristic (ROC) curve using “plotROC” package by R language. Finally, the expression of GLS was verified in the GSE62646 data set.

### The gene set enrichment analysis of GLS

The GSEA was analyzed based on GLS expression by the “clusterProfiler 4.0” package (R v4.1.2) between the high and low expression groups. *P* < 0.05 indicated a significant difference. The main differential signaling pathways associated with AMI were visualized.

### GLS expression in single-cell sequencing

The GSE184073 data set was evaluated using R language. The main steps are as follows: (1) construct a Seurat object through “Seurat” (30) R Package; (2) quality control (QC) of the raw data was performed by calculating the proportion of mitochondrial genes and ribosomal genes, and excluding low-quality cells; (3) the top 2,500 highly variable feature RNAs were filtered by the “FindVariableFeatures” function; (4) principal component analysis (PCA) and t-distributed stochastic neighbor embedding (tSNE) were performed for dimension reduction, clustering, and visualization based on the top 2,500 highly variable genes; (5) the R package “SingleR” (31) was used to annotate the single-cell RNA seq data automatically; (6) the expression data of GLS in different cells were extracted, and the differences were analyzed and visualized by the “ggplot2” R language package.

### Statistical analysis

Differences between the two groups were analyzed by Wilcoxon tests (mean ± SD), and *P* < 0.05 indicated statistical significance (ns: no significance, *P < 0.05, ^**^P < 0.01, ^***^P < 0.001, ^****^P < 0.0001). Kaplan–Meier analysis was implemented through the “tinyarray” package in R, and log-rank *P* < 0.05 was considered statistically significant.

## Results

### Differential expression landscape of cuproptosis-related genes

**FIGURE 1:**
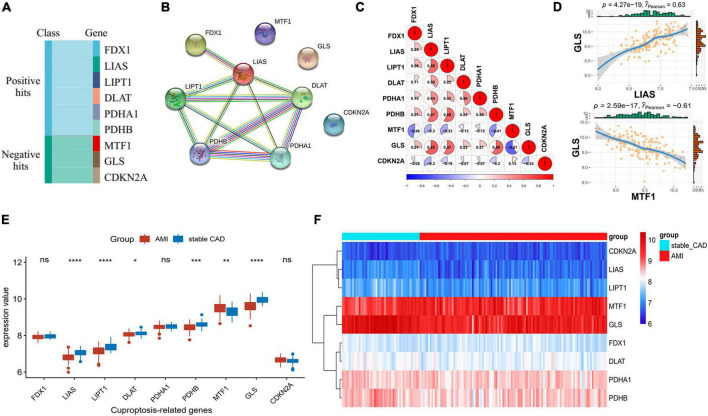
Differential expression landscape of cuproptosis-related genes (CRGs). **(A)** Classification of genes associated with CRGs. **(B)** Protein–protein interaction (PPI) network of CRGs. **(C,D)** Correlation analysis of CRGs in AMI. **(E)** The boxplot of CRGs differential expression. **(F)** The heatmap of CRGs (stable_CAD, stable coronary heart disease; AMI, acute myocardial infarction). *P < 0.05, **P < 0.01, ***P < 0.001, ****P < 0.0001.

Cuproptosis-related genes were garnered from previous literature (17). According to the report, FDX1, LIAS, LIPT1, DLAT, PDHA1, and PDHB were positively correlated with cuproptosis, but MTF1, GLS, and CDKN2A were the opposite (Figure 1A). PPI network of CRGs was constructed by STRING database, and the protein interaction was enriched (*P* = 1.36e-10), with an average node degree of 2.44 and an average local clustering coefficient of 0.622. We discovered that the positively correlated proteins were closely correlated, while the negatively correlated proteins were unrelated to others (Figure 1B). Next, we investigated the correlation of CRGs in the GSE59867 data set. The results indicated that there was a close correlation between them (Figure 1C; Supplementary Table 1). We also found that GLS had the largest positive correlation with LIAS (*P* = 4.27e-19, *r* = 0.63) and the largest negative correlation with MTF1 (*P* = 2.59e-17, *r* = −0.61), suggesting that it plays an important role in the above relationship (Figure 1D). The results of differential analysis showed that six (LIAS, LIPT1, DLAT, PDHB, MTF1, and GLS) of the nine CRGs were significantly different between the stable_CAD and AMI groups (Figure 1E). Compared with stable_CAD group, LIAS, LIPT1, DLAT, PDHB, and GLS were lower and MTF1 was higher in AMI. Notably, FDX1 was vital for cuproptosis, that is, no significant difference between the two groups. Based on gene expression values, CRGs were clustered into four groups by heatmap analysis, with higher GLS and MTF1 expression and lower CDKN2A, LIAS, and LIPT1 expression in all samples (Figure 1F).

### Functional enrichment analysis of cuproptosis-related genes

**FIGURE 2:**
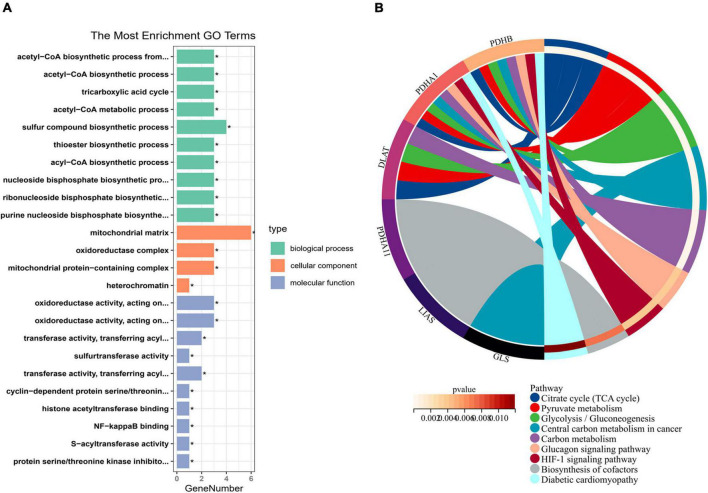
Enrichment analysis of cuproptosis-related genes (CRGs). **(A)** The histogram of GO analysis (BP, CC, and MF). **(B)** The Circos of Kyoto Encyclopedia of Genes and Genomes (KEGG) analysis. *P < 0.05.

To explore the regulatory mechanism of CRGs, we executed GO and KEGG enrichment analyses using R software. In biological process (BP) analysis (Figure 2A), these genes were mostly enriched in acetyl-CoA biosynthetic process from pyruvate, acetyl-CoA biosynthetic process, tricarboxylic acid (TCA) cycle, acetyl-CoA metabolic process, and sulfur compound biosynthetic process. In cellular component (CC) analysis (Figure 2A), these genes were mostly enriched in mitochondrial matrix, oxidoreductase complex, mitochondrial protein-containing complex, and heterochromatin. In molecular function (MF) analysis (Figure 2A), these genes were mostly enriched in oxidoreductase activity, acting on the aldehyde or oxo group of donors, NAD or NADP as acceptor, oxidoreductase activity, acting on the aldehyde or oxo group of donors, transferase activity, transferring acyl groups other than amino-acyl groups, sulfurtransferase activity, transferase activity, and transferring acyl groups. KEGG enrichment analyses showed that TCA cycle, pyruvate metabolism, glycolysis/gluconeogenesis, central carbon metabolism in cancer, carbon metabolism, glucagon signaling pathway, HIF-1 signaling pathway, biosynthesis of cofactors, and diabetic cardiomyopathy were mostly enriched (Figure 2B).

### The results of correlation analysis between cuproptosis-related genes and N6-methyladenosine-related genes

**FIGURE 3:**
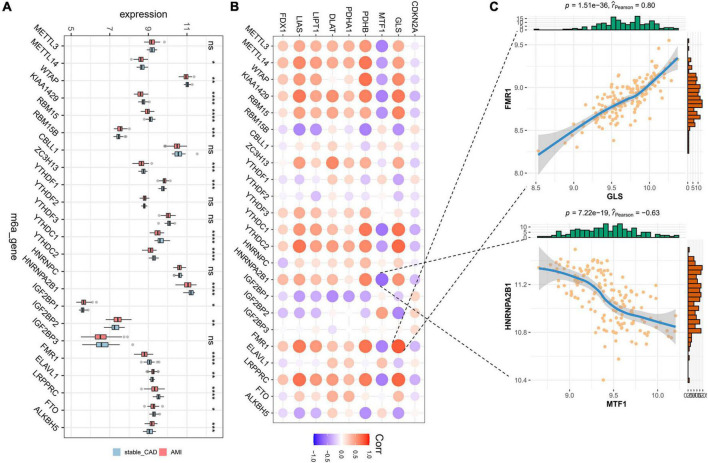
Landscape of m6A-related genes and the correlation degree between cuproptosis-related genes (CRGs) and m6A-related genes. **(A)** The boxplot of differential expression of m6A-related genes in AMI (stable_CAD, stable coronary heart disease; AMI, acute myocardial infarction). **(B)** The heatmap of the correlation degree between CRGs and m6A-related genes (the darker the color and the larger the graph, the more relevant it is. Red is positive, and purple is negative). **(C)** Correlation between GLS and FMR1 as well as MTF1 and HNRNPA2B1. *P < 0.05, **P < 0.01, ***P < 0.001, ****P < 0.0001.

Initially, we calculated the different expression of m6A-related genes between the stable_CAD and AMI groups. As previously reported (32–34), most m6A-related genes were significantly different between the two groups (Figure 3A), such as KIAA1429, RBM15, HNRNPA2B1, and FMR1, implying that m6A may be involved in the pathogenesis of AMI. We also investigated the relationship between CRGs and m6A-related genes. The results showed that there was a close relationship between them (Figure 3B; Supplementary Table 2). In this relationship network, GLS had the largest positive correlation with FMR1, and MTF1 had the largest negative correlation with HNRNPA2B1 (Figure 3C).

### The correlation analysis results of cuproptosis-related genes and immune characteristics in acute myocardial infarction

**FIGURE 4:**
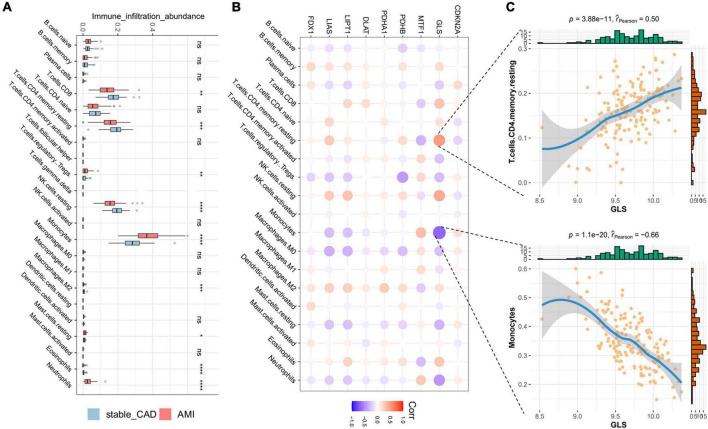
Landscape of immune cells and the correlation degree between cuproptosis-related genes (CRGs) and immune cells. **(A)** The boxplot of differential expression of immune cells in AMI (stable_CAD: stable coronary heart disease, AMI: acute myocardial infarction). **(B)** The heatmap of the correlation degree between CRGs and immune cells (the darker the color and the larger the graph, the more relevant it is. Red is positive, and purple is negative). **(C)** Correlation between GLS and T cells. CD4 memory resting as well as monocytes. *P < 0.05, **P < 0.01, ***P < 0.001, ****P < 0.0001.

In our study, the immune infiltration abundance was calculated by “CIBERSORT” algorithm, and we found that T cells CD8, T cells CD4 naive, T cells CD4 memory resting, resting NK cells, and monocyte immune infiltrating abundance were higher (Figure 4A). The immune infiltration abundance of nine immune cells was significantly different between the two groups (Figure 4A). Correlation analysis revealed that GLS was closely associated with a variety of immune cells (Figure 4B; Supplementary Table 3), such as T cells CD4 memory resting (*P* = 3.88e-11, *r* = 0.50) and monocytes (*P* = 1.1e-20, *r* = -0.66) (Figure 4C).

### The construction of competing endogenous RNA network, TF_mRNA network, and tiny molecule compound identification

**FIGURE 5:**
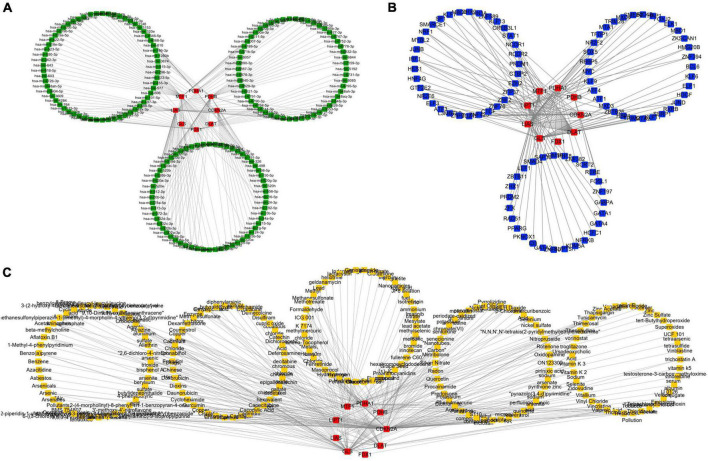
Regulatory mechanism of cuproptosis-related genes (CRGs). **(A)** The competing endogenous RNA (ceRNA) network of CRGs. **(B)** The TF network of CRGs. **(C)** The chemicals network of CRGs (Red nodes represent CRGs, green nodes represent miRNAs, blue nodes represent TF, and yellow nodes represent chemicals).

NetworkAnalyst was used to analyze ceRNA, TF, and tiny molecule compound identification. The ceRNA network consists of 183 nodes and 213 edges and contains eight CRGs and 175 miRNAs (Figure 5A; Supplementary Table 4). In this network, six CRGs (CDKN2A, DLAT, FDX1, LIAS, MTF1, and PDHB) had close interactions by targeting miRNAs. The TF_mRNA network consists of 102 nodes and 126 edges and contains nine CRGs and 93 TFs (Figure 5B; Supplementary Table 5). The protein–chemical interactions network consists of 218 nodes and 304 edges and contains nine CRGs and 209 chemicals (Figure 5C; Supplementary Table 6). Based on the degree of chemicals in this network, the top 30 were exhibited in Table 2.

**TABLE 2:** Top 30 chemicals.

Id	Label	Degree	Betweenness	Gene
D014635	Valproic acid	8	1991.61	CDKN2A, DLAT, FDX1,GLS, LIPT1, MTF1, PDHA1, PDHB
D019327	Copper sulfate	6	1203.07	CDKN2A, DLAT, GLS, LIPT1, MTF1, PDHB
D005557	Formaldehyde	5	424.62	DLAT, GLS, LIPT1, MTF1, PDHA1
D008741	Methyl methanesulfonate	4	231.97	DLAT, GLS, PDHA1, PDHB
D016572	Cyclosporine	4	218.89	FDX1,GLS, LIPT1, PDHB
D000082	Acetaminophen	4	214.27	DLAT, GLS, LIAS, PDHA1
C045651	Epigallocatechin gallate	3	512.85	CDKN2A, GLS, PDHB
C017947	Sodium arsenite	3	493.75	CDKN2A, MTF1, PDHB
D016604	Aflatoxin B1	3	485.68	CDKN2A, GLS, MTF1
D001564	Benzo(a)pyrene	3	456.5	CDKN2A, FDX1, GLS
C006632	Arsenic trioxide	3	386.35	CDKN2A, GLS, PDHA1
D014212	Tretinoin	3	386.35	CDKN2A, GLS, PDHA1
D002104	Cadmium	3	340.69	CDKN2A, MTF1, PDHA1
C059514	Resveratrol	3	340.69	CDKN2A, MTF1, PDHA1
C459179	4-(5-benzo(1,3)dioxol-5-yl-4-pyridin-2-yl-1H-imidazol-2-yl)benzamide	3	313.42	CDKN2A, DLAT, GLS
C516138	(6-(4-(2-piperidin-1-ylethoxy)phenyl))-3-pyridin-4-ylpyrazolo(1,5-a)pyrimidine	3	313.42	CDKN2A, DLAT, GLS
D002945	Cisplatin	3	313.42	CDKN2A, DLAT, GLS
C561695	(+)-JQ1 compound	3	313.42	CDKN2A, DLAT, GLS
D012822	Silicon dioxide	3	269.46	DLAT, GLS, MTF1
D001151	Arsenic	3	258.54	CDKN2A, DLAT, GLS
D011794	Quercetin	3	258.54	CDKN2A, GLS, LIPT1
D003300	Copper	3	204.64	CDKN2A, LIPT1, MTF1
D019284	Thapsigargin	3	159.1	GLS, LIAS, PDHB
D001280	Atrazine	3	47.22	LIAS, LIPT1, PDHA1
C006253	Pirinixic acid	2	290.23	CDKN2A, LIAS
C006780	Bisphenol A	2	188.17	CDKN2A, GLS
D004958	Estradiol	2	188.17	CDKN2A, GLS
D006861	Hydrogen peroxide	2	188.17	CDKN2A, GLS
C042720	mercuric bromide	2	188.17	CDKN2A, GLS
D010662	Phenylmercuric acetate	2	188.17	CDKN2A, GLS

### Prognostic value analysis of cuproptosis-related genes

**FIGURE 6:**
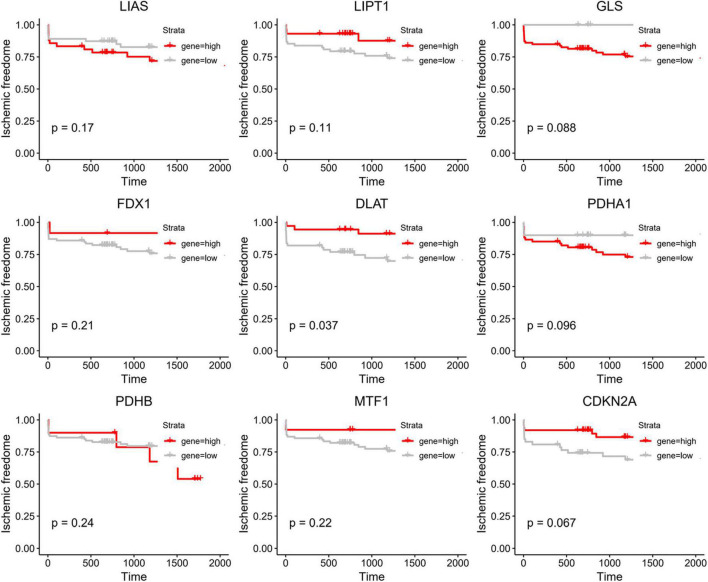
Kaplan–Meier curve of cuproptosis-related genes (CRGs).

To further investigate the role of CRGs in AMI, we utilized Kaplan–Meier methods to evaluate their prognostic value in the GSE21545 data set. We discovered that only DLAT had some prognostic value (Figure 6). Patients with high DLAT expression had higher ischemic freedom, suggesting that DLAT had a protective effect on ischemic events.

### Diagnostic value of cuproptosis-related genes

**FIGURE 7:**
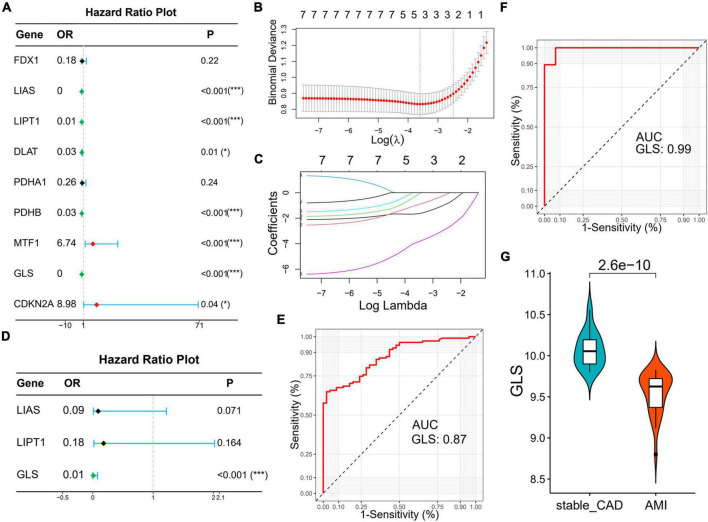
Identification of diagnostic markers for AMI based on logistic regression and lasso analysis. **(A)** The forest map of univariate logistic regression. **(B)** Log (Lambda) value of the seven genes in the lasso model. **(C)** The most proper log (Lambda) value in the lasso model. **(D)** The forest map of multivariate logistic regression. **(E,F)** The ROC curve of GLS in the training set and verification set, respectively. **(G)** The violin diagram of GLS differential expression is in the validation set (stable_CAD, stable coronary heart disease; AMI, acute myocardial infarction). *P < 0.05, ***P < 0.001.

Subsequently, we explored the diagnostic value of CRGs. Univariate logistic regression results showed that LIAS (OR: 0, 95% CI: 0–0.03, *P* < 0.001), LIPT1 (OR: 0.01, 95% CI: 0–0.06, *P* < 0.001), DLAT (OR: 0.03, 95% CI: 0–0.48, *P* = 0.01), PDHB (OR: 0.03, 95% CI: 0–0.18, *P* < 0.001), and GLS (OR: 0, 95% CI: 0–0.01, *P* < 0.001) were protective factors for AMI, while MTF1 (OR: 6.74, 95% CI: 2.09–21.76, *P* < 0.001) and CDKN2A (OR: 8.98, 95% CI: 1.15–70.36, *P* < 0.04) were risk factors for AMI (Figure 7A). Lasso analysis showed that three genes (LIAS, LIPT1, and GLS) were significantly different (Figures 7B,C). Multivariate logistic regression results suggested that only GLS was the protective factor for AMI (Figure 7D). According to ROC curve results, GLS could effectively distinguish AMI from stable_CAD (Figure 7E). The external data set validation results were consistent with the training set results (Figures 7F,G).

### Gene set enrichment analysis results

**FIGURE 8:**
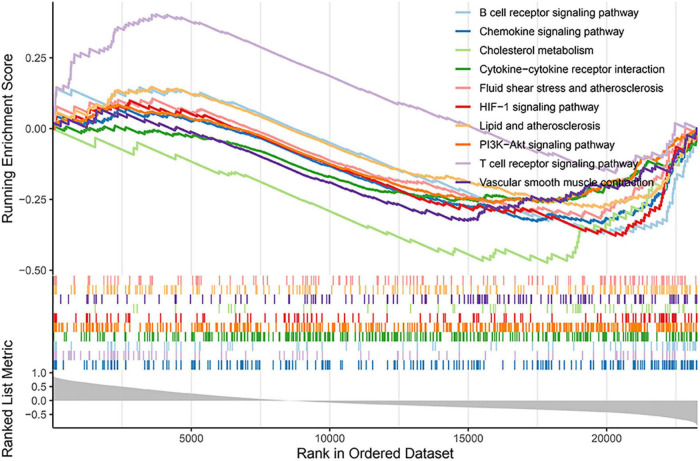
Gene set enrichment analysis (GSEA) results based on GLS expression.

Gene set enrichment analysis was used to further clarify the signaling pathways related to GLS expression. The results revealed that ribosome, herpes simplex virus 1 infection, spliceosome, and nucleocytoplasmic transport signaling pathways were enriched in the high expression group, while neuroactive ligand–receptor interaction, lysosome, neutrophil extracellular trap formation, osteoclast differentiation signaling pathways, tuberculosis, and parathyroid hormone synthesis, secretion, and action were enriched in the low expression group (Figure 8; Supplementary Table 7).

### The results of GLS expression in single-cell sequencing

**FIGURE 9:**
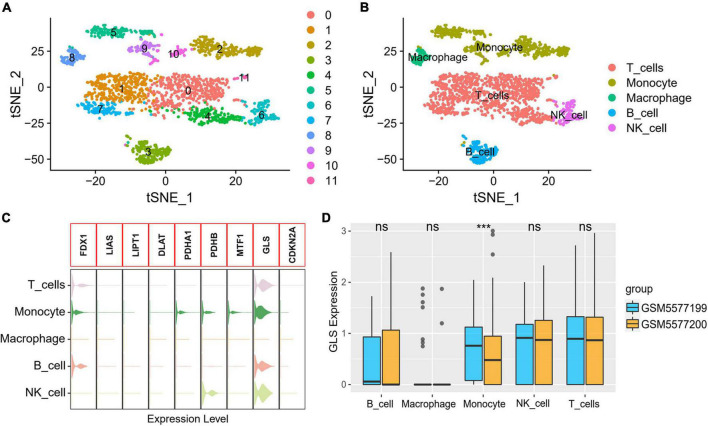
GLS expression in single-cell data. **(A)** Single-cell sequencing data dimensionality reduction, clustering results. **(B)** t-Distributed stochastic neighbor embedding (tSNE) visualization of cell annotation results. **(C)** Expression of CRGs in different cells. **(D)** Differential expression of GLS in five myeloid cells between two groups. ***P < 0.001.

A total of 23,320 genes and 2,223 cells were extracted from the GSE184073 data set after QC filtering. After reduction and clustering, these cells were divided into 12 clusters (Figure 9A). By further cell annotation, we obtained five types of myeloid cells (Figure 9B). In addition, we discovered that GLS is expressed in a variety of myeloid cells, excluding macrophages (Figure 9C). Finally, the differential analysis demonstrated that GLS expression in monocytes was significantly reduced in acute coronary syndrome (ACS) compared with stable angina pectoris (SAP) (Figure 9D).

## Discussion

In 2022, Tsvetkov et al. (17) reported cuproptosis, a form of cell death that is dependent on mitochondrial respiration, but there has been very little research on it in diseases. For the first time, we explore the diagnostic and prognostic value of CRGs and their possible regulatory mechanisms in AMI based on bioinformatics.

First, we performed PPI analysis and gene correlation analysis on CRGs. The results revealed that there was a close relationship between genes positively related to cuproptosis at the protein level. However, at the gene level, all CRGs were closely correlated with AMI. This suggests that there is a heterogeneity in the interactions of CRGs at the protein level and gene level or in different diseases. It was noteworthy that GLS had the largest positive correlation with LIAS and the largest negative correlation with MTF1, which is different from previous reports (17). We speculate that GLS may play different roles in different diseases. Second, we investigated the differences of CRGs between stable_CAD and AMI groups. The results showed that all genes except FDX1, PDHA1, and CDKN2A were significantly dysregulated between the two groups, implying that CRGs may be involved in the occurrence and development of AMI.

Macrophages play an important role in AMI. one–three days after the occurrence of AMI, macrophages derived from monocytes are recruited to the area of injury, and AM1 macrophages with pro-inflammatory effects produce inflammatory response to remove necrotic cell debris (35); then, between 4 and 7 days, M2 macrophages with repair function are used for tissue repair (36). During this process, macrophage polarization and inflammatory response directly alter the prognosis of patients. Studies have disclosed that energy metabolism of macrophages could regulate their polarization and inflammatory responses (37). M1 macrophages preferentially performed glycolysis, enhanced glycolysis derived higher inflammatory responses (38, 39), and inhibited glycolysis suppressed the increase of pro-inflammatory factors. However, M2 macrophages were preferred for TCA metabolic pathway. In conclusion, the regulation of macrophage energy metabolism can regulate macrophage polarization and inflammatory response. KEGG enrichment results indicated that CRGs were mainly involved in energy metabolism pathways such as TCA cycle, pyruvate metabolism, glycolysis/gluconeogenesis and carbon metabolism, and hypoxia-related pathways, such as HIF-1 signaling pathway. The results suggest that these metabolic pathways are expected to be therapeutic targets for AMI.

So far, there is increasing evidence that m6A is involved in the occurrence, development, and prognosis of AMI (32, 40, 41). A study showed that m6A level increased in mice at 4 weeks after AMI, and overexpression of FTO had a cardioprotective effect on AMI mice (32). In addition, FTO significantly reduced fibrosis and scar area in AMI mouse models, which were critical for preventing heart failure after AMI. Another study in a large sample of CAD patients and controls found that 304 m6A-SNPs were associated with CAD (40). The methylation level of m6A mRNA was increased in cardiomyocytes treated with hypoxia/reoxygenation and in IR-treated mice (41). Silencing METTL3 could reduce IR damage by enhancing autophagy and inhibiting apoptosis of H/R-treated credits, while ALKBH5 has the opposite effect to METTL3 (41). On the contrary, HNRNPA2B had been shown to regulate miRNA maturation and variable splicing in the nucleus (42). Recent research has found that FMR1 regulated RNA decay in an m6A-dependent manner (43). In summary, m6A plays an important role in various stages of AMI. Our study showed that there was a close interaction between CRGs and m6A-related genes, especially GLS and FMR1, MTF1, and HNRNPA2B.

Kaplan–Meier analysis showed that DLAT had prognostic value in ischemic events. Dihydrolipoamide S-acetyltransferase (DLAT) is an E2 subunit of the pyruvate dehydrogenase complex (PDC), which is a key enzyme in the catabolic glucose pathway (44). Studies had shown that DLAT was related to cell proliferation, and knocking down DLAT would inhibit cell proliferation by increasing intracellular pyruvate content (45). Moreover, compared with obese patients, DLAT expression was higher in the adipose tissue of healthy patients (46).

To gauge the diagnostic value of CRGs in AMI, COX combined lasso regression algorithm was used. The results showed that only GLS had diagnostic value. GLS, a substance essential for cellular energy metabolism, is responsible for the conversion of glutamine to glutamate. It converts glutamine into stoichiometric ammonia and glutamate after entering the mitochondria through glutamine transporters (47). Glutamate is used to produce adenosine triphosphate (ATP) by TCA cycle or for the synthesis of other amino acids and lipids, which are necessary for bioenergetics and biosynthesis (48). Up till the present moment, research on GLS has focused on oncology (49–51); however, the role in AMI is unclear. Our study suggests that GLS is a promising therapeutic target for AMI.

The ceRNA analysis revealed that eight CRGs (CDKN2A, DLAT, FDX1, GLS, LIAS, MTF1, PDHA1, and PDHB) and 175 miRNAs networks were constructed. Moreover, we analyzed GLS-related miRNAs through literature retrieval. We found that miR-23a-3p and miR-7-5p have been studied in AMI (52–54), but the regulatory mechanism of miR-335-5p in AMI remains unclear. Some studies have shown that miR-23a-3p could be used as a biomarker for ST-segment elevation AMI (52). Subsequently, mechanistic studies suggested that miR-23a-3p knockdown inhibited myocardial injury by curbing ferroptosis (54). Other studies had shown that miR-7-5p regulated the injury of hypoxic cardiomyocytes, and miR-7-5p upregulation promoted the injury of hypoxic H9c2 cells (53, 55). Therefore, the role of Mir-335-5p is worthy of further exploration in AMI.

Immune landscape and single-cell sequencing analysis indicated that GLS was closely related to monocytes. The research indicated that monocyte recruitment blocking was beneficial for the treatment of AMI, and the mechanism was mainly by inhibiting the inflammatory reaction (56). Our study discovered that GLS was negatively correlated with monocyte immune infiltration, and GLS expression was significantly decreased in ACS. We speculate that GLS may play a role in inhibiting monocyte infiltration and thus play an anti-AMI role.

Finally, we also investigated the biological functions and regulatory mechanisms of GLS. Interestingly, GSEA demonstrated that GLS was involved in immune-related signaling pathways, such as T-cell receptor signaling pathway and chemokine signaling pathway. Moreover, GLS was also involved in hypoxia-related pathways, such as HIF-1 signaling pathway, which was consistent with the analysis results of KEGG. Increasing evidence has indicated that immune response and hypoxia play an important role in the occurrence and development of AMI (21, 57, 58). For example, CD4 + T cells promoted myocardial ischemia–reperfusion injury through IFN-γ expression (59); CD8 + T cells induced by cytomegalovirus (CMV) infection participated in acute coronary events (60, 61); macrophage Smad3 signaling stimulated phagocytosis and regulated inflammation, which protected the infarction heart, reduced mortality (62); chemokines, such as CCL2 and CXCL12, were involved in cardiac injury, repaired and remodeled by dominating the inflammatory cascade (63, 64). Moreover, from the current experimental evidence, the activation and inactivation of HIF-1 signaling pathway played a protective role in the heart by participating in the regulation of apoptosis in injured regions (65). Therefore, we hypothesized that GLS may be involved in AMI by regulating the immune microenvironment through HIF-1 signaling pathway.

## Conclusion

In conclusion, this research systematically investigated the molecular changes and interactive gene landscape of cuproptosis in AMI. Our research indicates that these CRGs may play a key role in the diagnosis and prognosis of AMI. GLS is likely to participate in the occurrence and development of AMI through the HIF-1 signaling pathway. However, the mechanism of GLS involvement in AMI still warrants further verification at the human, animal, and cellular levels. Even so, our results provide new insights into the diagnosis, prognosis, and therapy of AMI.

## Data availability statement

The original contributions presented in the study are included in the article/Supplementary material, further inquiries can be directed to the corresponding author.

## Ethics statement

Ethical review and approval was not required for the study on human participants in accordance with the local legislation and institutional requirements. Written informed consent from the patients/participants or patients/participants’ legal guardian/next of kin was not required to participate in this study in accordance with the national legislation and the institutional requirements.

## Author contributions

ZL, LW, and QX were responsible for the design project. ZL, LW, YH, WL, and QY analyzed the data and visualized them. ZL, RL, and NH wrote and edited the manuscript. LW, QX, and XL were responsible for reviewing and correcting the manuscript. All authors contributed to the article and approved the submitted version.

## Footnotes

1
https://www.ncbi.nlm.nih.gov/geo/

2
https://cn.string-db.org/

3
https://www.networkanalyst.ca/

## Abbreviations

AMI: acute myocardial infarction
CRGs: cuproptosis-related genes
stable_CAD: stable coronary heart disease
m6A: N6-methyladenosine
GO: gene ontology
BP: biological process
CC: cellular component
MF: molecular function
KEGG: Kyoto Encyclopedia of genes and genomes
AUC: area under curve
ROC: receiver operating characteristic
GSEA: gene set enrichment analysis
GEO: gene expression omnibus database
PPI: protein–protein interaction
TCA: tricarboxylic acid
ceRNA: competing endogenous RNA
TF: transcription factor
ATP: adenosine triphosphate
CMV: cytomegalovirus
CIBERSORT: cell-type identification by estimating relative subsets of RNA transcript
QC: quality control
PCA: principal component analysis
tSNE: t-distributed stochastic neighbor embedding
ACS: acute coronary syndrome
SAP: stable angina pectoris.
